# Diagrammatic prevalence index: a new algorithm to evaluate pine wilt disease prevalence at the sub-compartment scale

**DOI:** 10.3389/fpls.2025.1578700

**Published:** 2025-07-09

**Authors:** Yanjun Zhang, Siyuan Zheng, Jinjuan Bai, Jiafu Hu, Yongjun Wang

**Affiliations:** ^1^ College of Forestry and Biotechnology, Zhejiang A&F University, Hangzhou, China; ^2^ China National Bamboo Research Center, Hangzhou, China; ^3^ Zhejiang Provincial Forest Disease and Pest Control Station, Hangzhou, China

**Keywords:** *Bursaphelenchus xylophilus*, epidemiology, diagrammatic scale, diagrammatic prevalence index, algorithm

## Abstract

Pine wilt disease (PWD), caused by the nematode *Bursaphelenchus xylophilus*, has led to significant ecological and economic losses in pine forests worldwide. Historically, several metrics, including the number of PWD-infected trees, the proportion of PWD-infected pine sub-compartments, and the occurrence area, have been employed to evaluate the prevalence of PWD. However, these metrics are individual and limited in comprehensively representing the prevalence of PWD in extensive regions. This study introduces a new algorithm for evaluating PWD prevalence in Hangzhou, China, where the disease has been established for over two decades. The algorithm utilizes data on the information of PWD-infected trees and sub-compartments to develop a diagrammatic scale (*DS*) and diagrammatic prevalence index (*DPI*). The *DS* categorizes the natural logarithm of the number of PWD-infected trees per hectare into 12 levels, providing a scale for semi-quantifying prevalence status within a sub-compartment. The *DPI* summarizes the occurrence and status of PWD-infected sub-compartments PWD in the geographic regions. The application of DPI in analysis of PWD prevalence in Hangzhou from 2021 to 2023 revealed consistent dynamic patterns of and accuracy, compared to other metrics. The *DS* and *DPI* might contribute to the improvement of accuracy, precision, reproducibility and repeatability of PWD prevalence assessment.

## 1 Introduction

Pine wilt disease (PWD) is a devastating forest disease caused by the nematode *Bursaphelenchus xylophilus*, which is primarily transmitted by the pine sawyer beetle, *Monochamus* spp (Mamiya, 1983; Futai, 2013). The disease, which originated in North America, has caused significant ecological and economic losses worldwide, particularly in East Asia, where it has led to the rapid decline and death of pine forests (Zhao et al., 2014; Back et al., 2024). This exotic pathogen has also recently spread to European countries, such as Portugal and Spain (Vicente et al., 2011). The etiology of PWD involves a complex interplay between the nematode, pine sawyer beetle, and the host pine tree (Zhao et al., 2014; Xu et al., 2023). The pine sawyer beetle serves as a vector for the nematode, facilitating its spread from infected to healthy trees (Jones et al., 2008; Akbulut and Stamps, 2012). The transportation of infected wood-containing pine sawyer beetles by humans can exacerbate the spread of the disease and lead to long-distance transmission (Rands et al., 2009). The rapid spread and high mortality rate of PWD pose serious challenges for forest management and ecological conservation efforts worldwide (Rodrigues, 2008; EPPO, 2012; Ye, 2019). China has the highest incidence of pine wilt disease worldwide. Since its introduction in 1982, PWD has spread rapidly in China and has led to the outbreaks in 19 provinces. Approximately 60,000,000 ha of pine forests in China are threatened by PWD (Xu et al., 2023). Therefore, accurate monitoring and effective management of PWD prevalence are urgently required.

Prevalence evaluation of PWD is essential for the development of effective management strategies (Rodrigues, 2008; Ye, 2019; Back et al., 2024). Historically, research has predominantly focused on evaluating the risk of PWD invasion into new ecological niches and predicting the pioneer areas for the spread of PWD on large geographic scales, such as climate conditions (Wong et al., 2017; Gruffudd et al., 2019; Tang et al., 2021) and stand structure (De La Fuente and Saura, 2021; Liu et al., 2023b; Schafstall et al., 2024). However, less attention has been directed toward the epidemiological patterns of PWD in areas where the disease has been established for extended periods.

For accurate prevalence evaluation of a plant disease, data collection at a fine geographic scale are required (Barzman et al., 2015; Firmino et al., 2017). Previous studies on PWD epidemiology have often relied on data at the county or municipal level (Lv et al., 2024; Schafstall et al., 2024). By contrast, data collection at finer geographic scales, such as townships and villages, would provide more detailed information for developing precise management strategies (Liu et al., 2023a). In Zhejiang Province, China, the prevalence data of PWD using unmanned aerial vehicles (UAVs) had been collected from 2021 to 2023, and all infected pine trees were precisely located by coordinates, making it feasible to conduct epidemiological analysis at a finer scale, such as at the sub-compartment scale (Zhang et al., 2024).

Quantification is essential for disease prevalence assessment and the efficient implementation of disease management programs (Nutter et al., 1991; Kwon et al., 2011; Bock et al., 2022). In the case of PWD, several metrics such as the number of PWD-infected trees, the proportion of PWD-infected pine sub-compartments, and the occurrence area, have been commonly employed in prevalence quantification (Hao et al., 2022). However, these metrics has limitations in comprehensively representing the prevalence of PWD, especially in in regions where PWD has been established for an extended period of time. Nevertheless, no studies on a finer geographic scale have been conducted to date. In this study, we introduced a new quantitative algorithm to develop new metric for PWD prevalence evaluation. This new metric integrated the information, such as the number of PWD-infected trees, the proportion of PWD-infected pine sub-compartments, and the occurrence area, which might contribute to the improvement of accuracy, precision, reproducibility and repeatability of PWD prevalence evaluation.

## 2 Materials and methods

### 2.1 The study area

**Figure 1:**
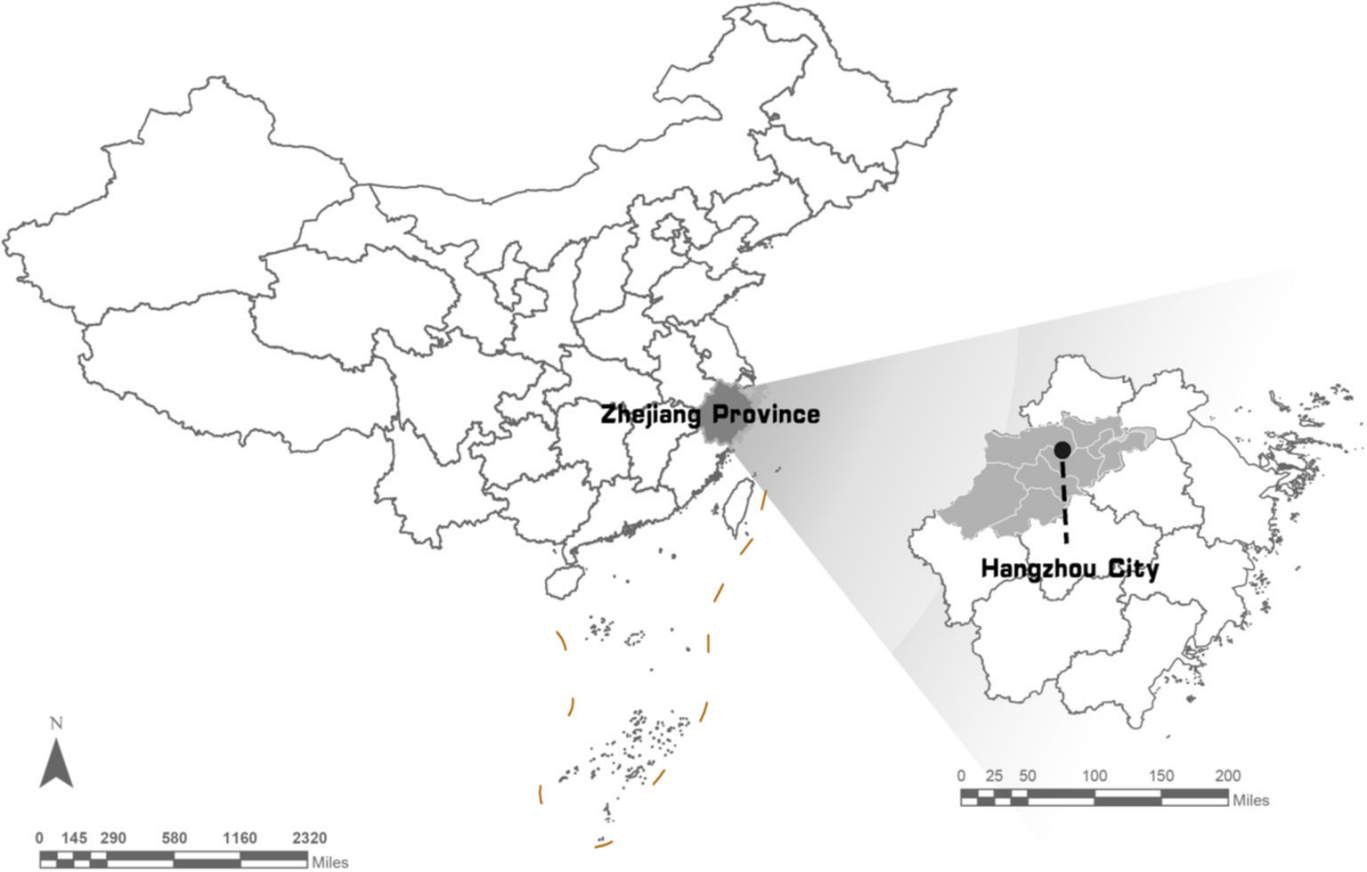
Study area for PWD surveillance at sub-compartment scale. All PWD-infected tree were located coordinately in the sub-compartments in the Digital Forest Protection system (https//szsf.lyj.zj.gov.cn).

The study area, Hangzhou, is a significant central city in the Yangtze River Delta in southeastern China (**Figure 1**). It is situated between 29°11’ and 30°34’ north latitude and 118°20’ and 120°37’ east longitude, encompassing a total area of 503,826 ha of pine forest. Masson pine (*Pinus massoniana*) is the dominant species. Hangzhou administers 7 pine-dominated county-level regions, including Xiaoshan distinct (XSQ), Yuhang distinct (XSQ), Fuyang distinct (XSQ), and Lin’an (XSQ), as well as two counties, Tonglu (TLX) and Chun’an (CAX), and one county-level city, Jiande (JDS). Topologically, the pine forest area in Hangzhou constitutes 24.3% of the total forest area, and the pine timber volume accounts for 37.5% of the total timber volume. PWD in Hangzhou was initially detected in Fuyang district in 1995, and has resulted in the mortality of numerous pine trees (Liu et al., 2001).

### 2.2 The sub-compartment design

A sub-compartment constitutes the smallest unit for forest management and organization of timber production, denoting a section with substantially uniform internal characteristics and distinct differences from adjacent areas (**Figure 1**) (Xie et al., 2011). It is also serves as a fundamental unit for forest resource investigation and management (Liu et al., 2023a). Within the work area, sub-compartments with identical site conditions, stand factors, cutting methods, management measures, and logging systems are aggregated. The delineation of the sub-compartment is primarily determined by the natural zoning of the logging system (Zhang et al., 2014). Typically, the area of a sub-compartment should be approximately 5 ha, with a maximum of 20 ha.

### 2.3 Data collection

This study utilized PWD occurrence data obtained in Hangzhou city, Zhejiang Province, China from 2021 to 2023. The sub-compartmental-level occurrence data of PWD were provided by the Zhejiang Provincial Forest Disease and Pest Control Station of China (ZJSF) and deposited in the Digital Forest Protection system (szsf.lyj.zj.gov.cn, accessed on 6 July 2024) (Zhang et al., 2024). Statistical data on the occurrence of PWD areas were collected by the forestry bureaus of various districts and counties in Zhejiang Province, based on ground and UAV investigation. The data were subsequently reported to the ZJSF and National Forestry and Grass Administration Forest and Grassland Pest Control Station of China (NFGA) through the autumn census each year. The data fields encompass the numbers of diseased and dead trees in every sub-compartment and the area of sub-compartment. Data were verified by the Quarantine Office of the Forest and Grassland Pest Control Station of the State Forestry and Grassland Administration.

### 2.4 Epidemiological parameters definition

Disease prevalence (*DP*) was calculated as the percentage of fields where the disease was detected (Nutter et al., 1991). To provide a more precise characterization of the PWD prevalence, epidemiological parameters based on the sub-compartmental scale were defined as follows:

1. *N*, Number of PWD-infected trees within each sub-compartment.
2. *NC*, The average number of PWD-infected trees per ha within one sub-compartment.
3. Ln (*N*), the natural logarithm value of *N*.
4. Ln (*NC*), the natural logarithm value of *NC*.
5. *DS*, Diagrammatic scale for PWD evaluation.
6. *DPI*, Diagrammatic prevalence index.

*DPI* was calculated based on *DS* scores in a certain geographic/administrative scale (Equation 1) (Chiang et al., 2017).


(1) $$DPI=\frac{\sum (DS\;\text{frequency}\times \text{score of}\;DS)\times 100}{\left(\text{Total number of}\;\text{SC})\times (\text{maximal }DS\right)}$$

### 2.5 Diagrammatic scale for evaluation of PWD prevalence at the sub-compartment scale

Based on the histogram distribution of the calculated PWD Ln (*NC*) values, intervals with higher Ln (*NC*) value were selected to establish the levels of the diagram. The diagrammatic scale (*DS*) was developed by utilizing the higher frequency class intervals of Ln (*NC*) values, and in the adapted Weber-Fechner law (Nutter and Schultz, 1995; Belan et al., 2014; Perina et al., 2019). Following the determination of the prevalence intervals to be represented and considered the shape and distribution of Ln (*NC*) values, a 12-intervals were employed to generate the *DS*.

### 2.6 Data analysis

The obtained data were analyzed using the IBM SPSS Statistics (version 20.0; SPSS Inc.), GraphPad Prism 9.5, and Microsoft Excel 2007. Histograms were used for investigating the distribution of PWD occurrence from the sub-compartment and to identify the shape of the distribution. The component statistics were compared among different epidemiological parameters methods, including mean (ma), median (md), standard deviation (σ), variance (σ^2^), skewness (Sk), and Kurtosis (bk).

## 3 Results

### 3.1 Baseline accuracy for PWD prevalence at the sub-compartment scale

**Figure 2:**
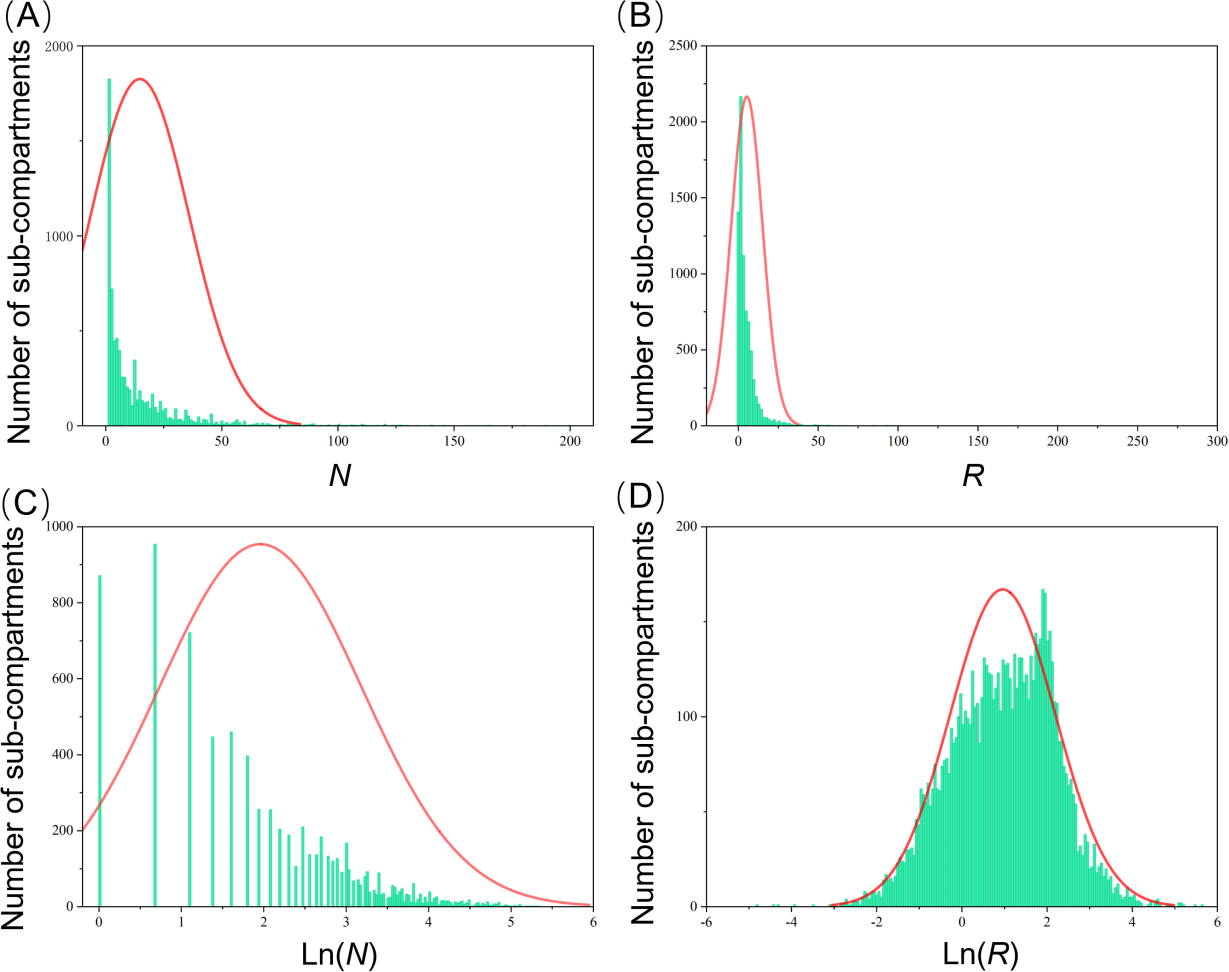
Histogram of occurrence of PWD based on *N* **(A)**, *NC* **(B)**, Ln (*N*) **(C)**, and Ln (*NC*) **(D)** in the sub-compartment level in Hangzhou in 2021.

Utilizing PWD prevalence data from 7,818 pine forest sub-compartments in Hangzhou, China, collected in 2021, we mapped the distributions of four key indexes, including *N*, *NC*, Ln (*N*), and Ln (*NC*), at the sub-compartment level (**Figure 2**). Our analysis revealed substantial variations in these distributions across the different prevalence index parameters (**Table 1**). Notably, the distribution of *N* exhibited a pronounced leftward skew, with a mean of 14.73 and a median of 7. The variance (σ^2^) was high at 449.98, accompanied by a skewness (Sk) of 3.49 and kurtosis (bk) of 16.93. In contrast, the *R* value displayed an even more pronounced leftward skew and kurtosis, with a variance (σ^2^) of 96.87, skewness (Sk) of 10.74, and kurtosis (bk) of 199.76. When comparing Ln (*N*) and Ln (*NC*) to *N* and *R*, we observed a significant normalization of skewness. Specifically, the variance (σ^2^), skewness (Sk), and kurtosis (bk) of Ln (*N*) decreased to 1.50, 0.15, and -0.77, respectively. However, Ln (*NC*) demonstrated the most optimal normal distribution, with an average of 0.96, a median of 1.04, a variance (σ^2^) of 1.53, a skewness (Sk) of -0.18, and a kurtosis (bk) of -0.05. Consequently, Ln (*NC*) was chosen as the baseline accuracy metric for further research into PWD prevalence dynamics at the sub-compartment scale.

**Table 1:** The statistical analysis of different epidemiological parameters.

Statistical analysis	*N*	*R*	Ln (*N*)	Ln (*NC*)
Mean (μ)	14.73	5.35	1.96	0.96
Median	7	2.82	1.95	1.04
Standard Deviation (σ)	21.21	9.84	1.22	1.24
Variance (σ^2^)	449.98	96.87	1.50	1.53
Skewness (*S_k_*)	3.49	10.74	0.15	-0.18
Kurtosis (bk)	16.93	199.76	-0.77	-0.05

### 3.2 Development of a diagrammatic scale to semi-quantifying PWD prevalence at the sub-compartment scale

**Figure 3:**
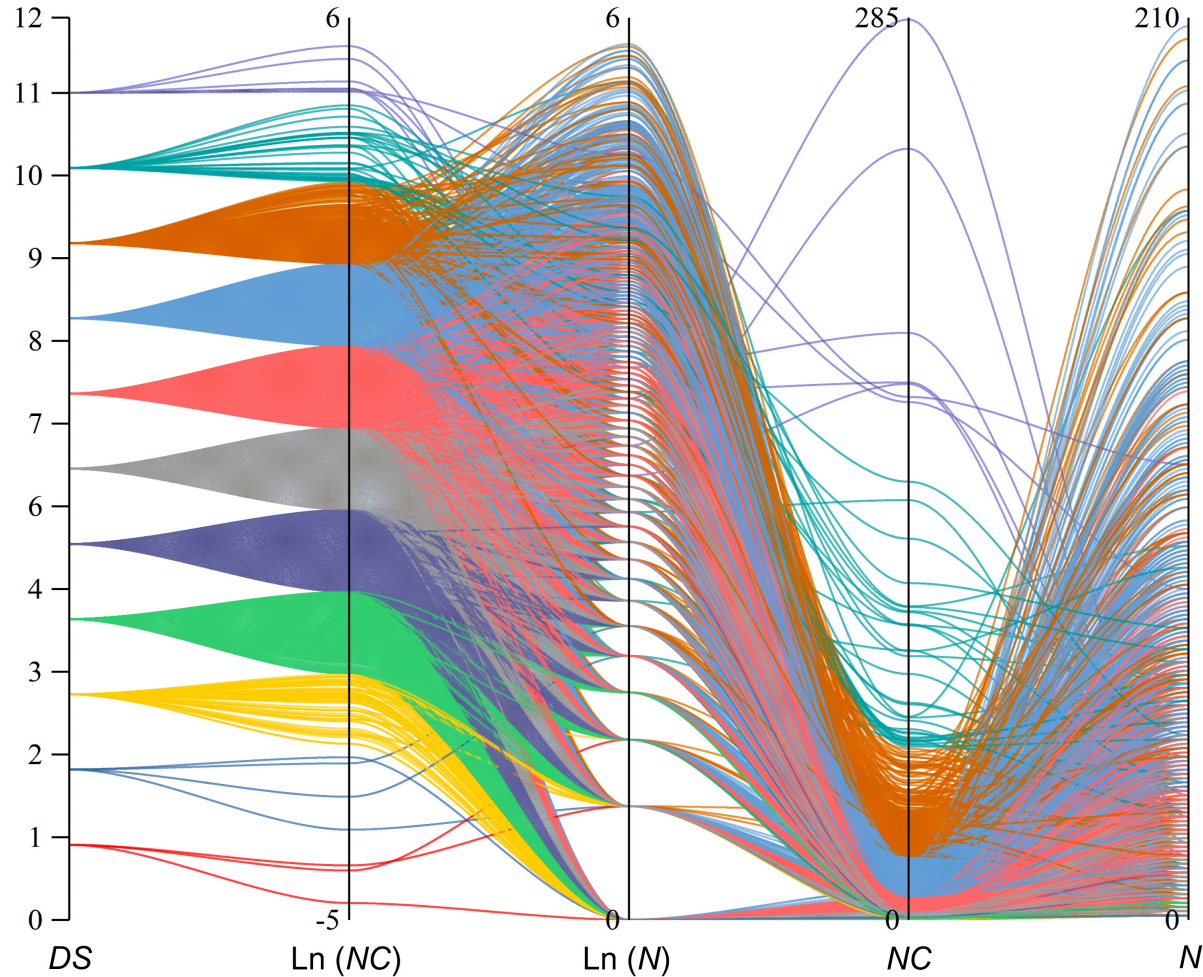
Parallel set plot for different prevalence indexes of 7,818 forest sub-compartments in 2021 Hangzhou, colours to differentiate diagrammatic scale (*DS*).

Utilizing the normal distribution of Ln (*NC*) values from PWD prevalence parameters at the sub-compartment scale in Hangzhou for 2021, we categorized these values into 12 levels (**Table 2**). For each Ln (*NC*) level, we established a corresponding diagrammatic scale (*DS*), with *DS* values ranging from 0 to 12 and linearly correlated with Ln (*NC*). A *DS* value of 0 indicates the absence of PWD-infected pine trees within the sub-compartments. Furthermore, we conducted a parallel aggregation analysis on the prevalence indices *N*, *R*, Ln(*N*), Ln (*NC*), and *DS* across all PWD-infected sub-compartments (**Figure 3**). The analysis revealed a normal distribution along the *DS* value axis, with concentrations predominantly at levels 5, 6, 7, and 8, comprising 90.32% of the total. This distribution pattern was also consistent in the Ln (*NC*) axis. Notably, significant crossover phenomena were observed along the Ln (*N*) axis, where lower *E* values appeared at higher positions on the Ln (*N*) axis. Similar crossover effects were observed for the *NC* and *N-* value axes.

**Table 2:** Diagrammatic scale (*DS*) set for pine wilt disease prevalence at the sub-compartment scale.

Diagrammatic scale, (*DS*)	Ln (*NC*) value	*NC* (*N* per ha)
1	Ln (*NC*) ≤ -5.00	*NC* ≤ 0.0067
2	-5.00 < Ln (*NC*) ≤ -4.00	0.0067 < *NC* ≤ 0.0183
3	-4.00 < Ln (*NC*) ≤ -3.00	0.0183 < *NC* ≤ 0.0183
4	-3.00 < Ln (*NC*) ≤ -2.00	0.0067 < *NC* ≤ 0.0498
5	-2.00 < Ln (*NC*) ≤ -1.00	0.0498 < *NC* ≤ 0.1353
6	-1.00 < Ln (*NC*) ≤ 0.00	0.1353 < *NC* ≤ 0.3679
7	0.00< Ln (*NC*) ≤ 1.00	0.3679 < *NC* ≤ 1.0000
8	1.00< Ln (*NC*) ≤ 2.00	1.0000 < *NC* ≤ 2.7183
9	2.00< Ln (*NC*) ≤ 3.00	2.7183 < *NC* ≤ 7.3891
10	3.00< Ln (*NC*) ≤ 4.00	7.3891 < *NC* ≤ 20.0855
11	4.00< Ln (*NC*) ≤ 5.00	20.0855 < *NC* ≤ 54.5982
12	5 < Ln (*NC*)	54.5982 < *NC*

### 3.3 Validation of the diagrammatic scale

**Figure 4:**
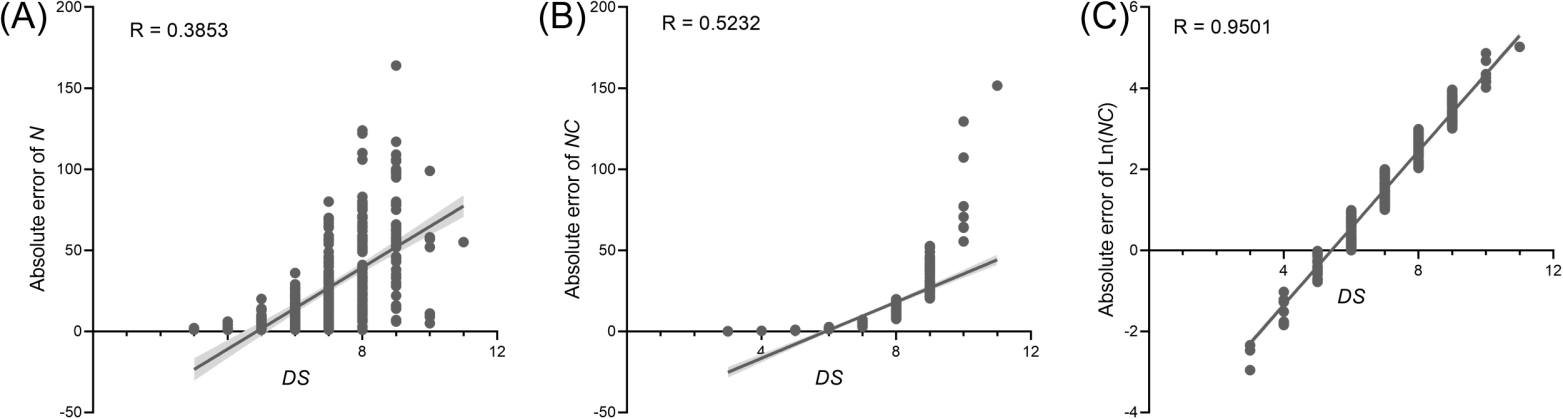
Relationship of *N* value **(A)**, R value **(B)**, and Ln (NC) value **(C)** to DS value based on 16 randomly selected pine wilt disease (PWD) prevalence at the village-level. The solid line represents the best fitting line. Box plot of coefficient of determination (R2) statistics for severity estimations by raters based on the linear regression relationship between different parameters and DS.

The diagrammatic scales were validated using the PWD prevalence data from 16 randomly selected villages. Linear regression analysis of these 16 randomly selected datasets revealed a positive linear relationship between various parameters, employing *N*, *NC*, or Ln (*NC*) and the *DS* value (**Figure 4**). However, the coefficient of determination (CoD. R^2^) exhibited a substantial variation. The regression R^2^ value for the relationship between *N* value and *DS* value was the lowest at 0.39. The R^2^ value for *NC* and *DS* was 0.52, whereas that for Ln (*NC*) and *DS* was the highest at 0.92.

### 3.4 Application of diagrammatic prevalence index for PWD prevalence in Hangzhou

**Figure 5:**
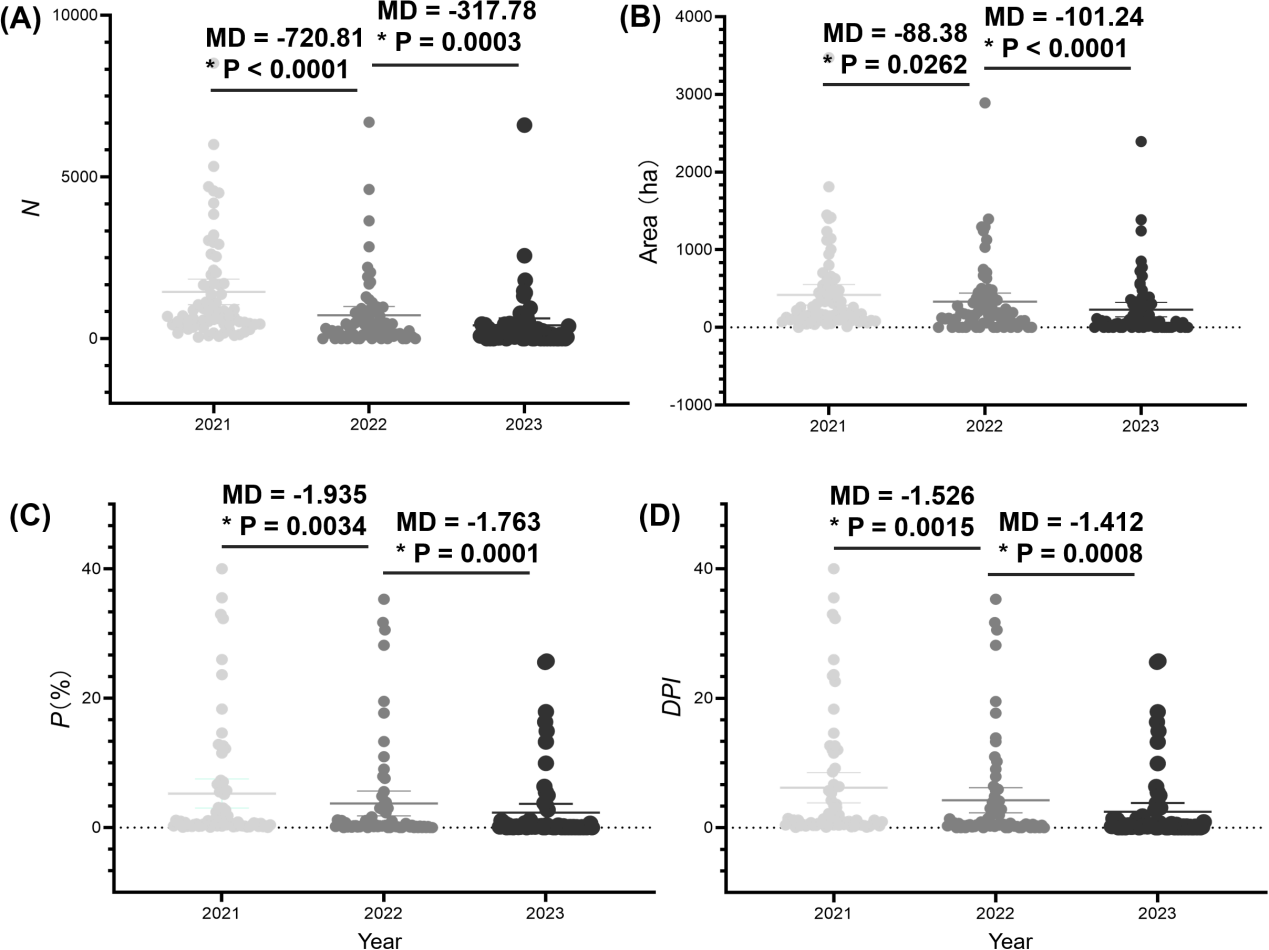
Analysis of the prevalence of pine wilt disease (PWD) in Hangzhou city from 2021 to 2023 based on the numbers of PWD-infected trees (*N*) **(A)**, PWD-infected sub-compartment area **(B)**, percentage of PWD-infected sub-compartment (*P*) **(C)**, and diagrammatic prevalence index (*DPI*) **(D)**. The solid circles represent individual metric values for each town. The central lines mark the mean value. The error bars across the boxes indicates the standard errors. MD indicates the difference of mean. Asterisks (*) in the figure indicate significant differences derived from two-sample comparisons (*P* = 0.05).

In conjunction with sub-compartment data from unaffected pine forests, we introduced the diagrammatic prevalence index DPI to summarize the PWD prevalence of PWD across the region. In 2021, Hangzhou had a total of 118,546 infected pine trees with *P* value of 13.32%, calculated with an *E* value of 7.18 (**Table 3**). In 2022, the total number of infected pine trees was 68,293 with *P* value of 11.17% and a DPI value of 5.67, representing decreases of 42.39%, 16.16%, and 20.94% compared to 2021, respectively. In 2023, the total number of infected pine trees was 39,061 with *P* value of 7.23%, and *DPI* value of 3.60, demonstrating decreases of 42.80%, 35.32%, and 36.55% respectively, compared to 2022. *DPI* index provides a novel metric for evaluating the prevalence in Hangzhou. Combined with *DS* value for each sub-compartment and the occurrence and status of PWD-infected sub-compartments, we calculated the diagrammatic prevalence indexes (*DPI*) of each town in Hangzhou from 2021 to 2023 (**Figure 5**). The analysis of *DPI* values from a total of towns revealed consistent dynamic patterns in PWD decline, aligning closely with the outcomes derived from other metrics. The near-identical annual reduction rates (~42%) suggest consistent efficacy of PWD-infected tree removal and sanitation. However, the sharper decline in DPI highlights its sensitivity to spatial clustering. As management prioritized high-*DS* sub-compartments (mainly levels 5–8), localized hotspots were neutralized, reducing regional aggregation.

**Table 3:** Different prevalence indexes of pine wilt disease in Hangzhou City based on *N*, *P*, Area, and *DPI* values.

Metric *	Year		
	2021	2022	2023
*N*	118546	68293	39061
Area (ha)	465883.8	370924.7	287229.7
Percentage (*P*, %)	13.32	11.17	7.23
*DPI*	7.18	5.67	3.60

*N represents the total number of infected trees, *P* represents the percentage occurrence of the PWD prevalence in sub-compartments, and *DPI* indicates diagrammatic prevalence index.

### 3.5 County-level variability of diagrammatic prevalence index

**Figure 6:**
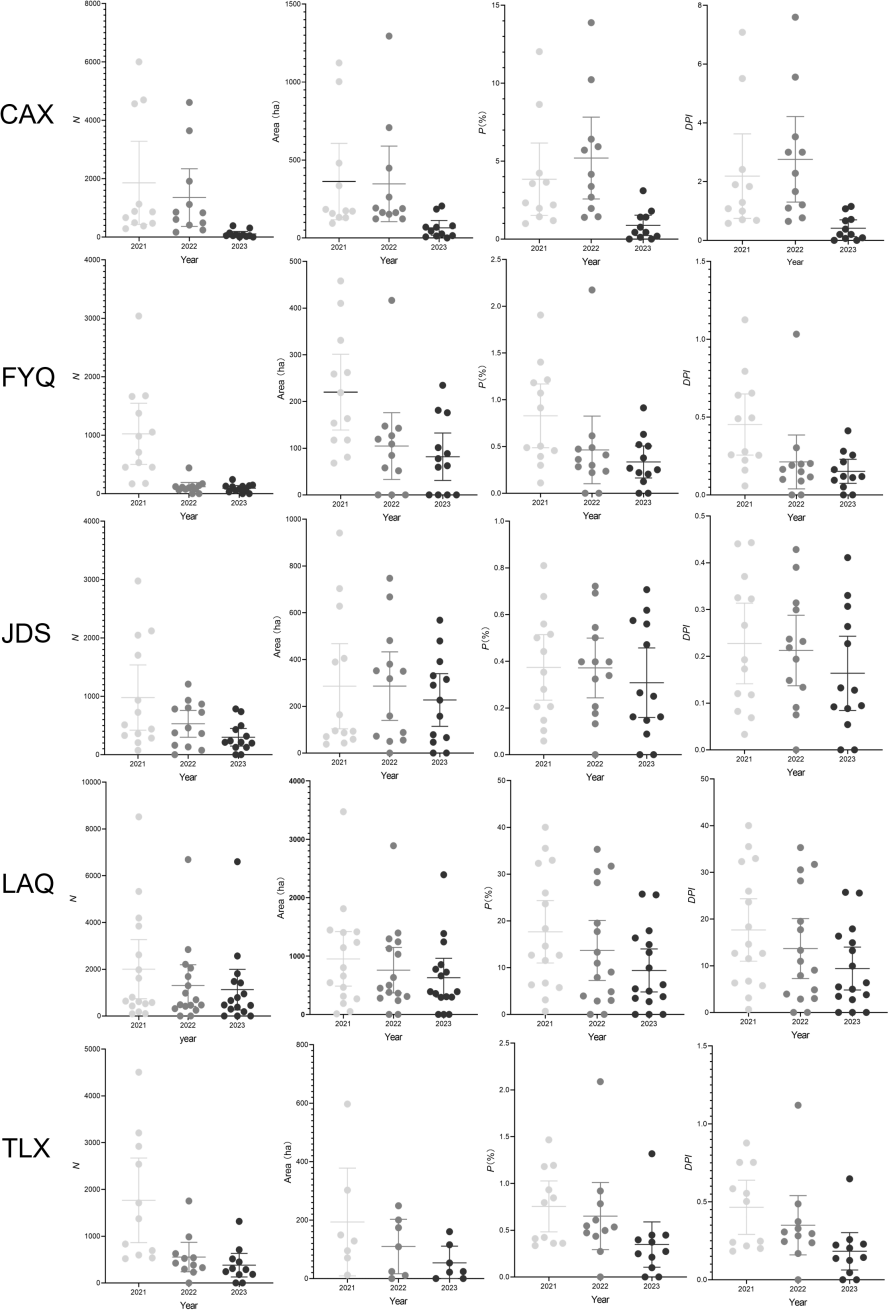
Analysis of the prevalence of pine wilt disease (PWD) in Chunan County (CAX), Fuyang County (FYQ), Jiande County (JDS), Linan County (LAQ), and Tonglu County (TLX) of Hangzhou city from 2021 to 2023 based on the numbers of PWD-infected trees (*N*), PWD-infected sub-compartment area, percentage of PWD-infected sub-compartment (*P*), and diagrammatic prevalence index (*DPI*). The solid circles represent individual metric values for each town. The central lines mark the mean value. The error bars across the boxes indicates the standard errors.

The study analyzed the prevalence of PWD at the county level from 2021 to 2023 using various metrics, including *DPI* (**Figure 6**). This variability was consistent with patterns observed using other metrics such as *N*, Area, and *P*. The results revealed significant variability in *DPI* across different counties. For example, faster declines of *DPI* in CAX correlate with the application of precise PWN management in 2023, while slower progress in FYQ reflects challenges in managing historically dense infestations.

## 4 Discussion

This study conducted a comprehensive analysis of the PWD prevalence in Hangzhou, China, in 2021, encompassing seven prevalence areas and 7,818 pine forest sub-compartments. A novel algorithm for semi-quantifying the PWD prevalence at the forest sub-compartment level was developed based on the number of infected pine trees and sub-compartment area. This innovative algorithm enables the quantitative evaluation of PWD prevalence across various geographic or administrative levels. In contrast to previous methods that relied on non-epidemic area risk models, this study’s algorithm is grounded in actual dataset from a PWD epidemic area with over 20 years of historical records (De La Fuente and Beck, 2018). The outcomes of this study provide multiple insights into the epidemiological patterns of PWD (Ikeda, 2007).

In recent decades, limitations in data acquisition and quantification have severely impeded the progress of PWD epidemiological studies in a fine geographic scale (Ecke et al., 2022). Since 2021, forestry authorities have determined the PWD prevalence data at the forest sub-compartment level to obtain a more precise understanding of PWD outbreaks in China. Forest sub-compartments are fundamental units for resource surveys and monitoring, and are essential for formulating forest management strategies, as well as for evaluation (Wu et al., 2012). Beginning in 2021, a digital forest protection platform was implemented in in Zhejiang Province, China. UAVs were used to conduct the PWD survey at the individual tree level, and the data within each forest sub-compartment were aggregated. This study represents the initial quantification of PWD incidence on a fine geographic scale, specifically in a sub-compartment scale. This approach offers the advantage of providing more accurate PWD occurrence data, thereby enabling forestry administrations to develop precise preventive and control plans.

Typically, quantification of plant disease severity relies on estimation methods based on visual assessments or image analysis of infected plant parts (Nutter and Schultz, 1995; Bock et al., 2020, 2022). However, in our study, we quantified PWD based on the determined data at the sub-compartment scale. Notably, different diseases may have distinct indices or assessment methods, and specific details can be found in relevant research papers or agricultural guidelines (Chiang et al., 2017; Madden et al., 2017). Furthermore, the accuracy and reliability of the plant disease index depend on the quality and consistency of data collection and analysis (Bock et al., 2010; Huang et al., 2024). The plant disease index is a crucial indicator used to evaluate the severity of plant diseases, usually calculated based on the degree of damage or infection observed in plants (Chiang et al., 2017). However, in PWD, infected pine trees invariably succumb to the disease. Therefore, when analyzing disease severity, we generally do not utilize the degree of damage to individual pine trees as the standard for disease severity; instead, we employ metrics such as the affected area and number of infected trees as indicators of disease severity or PWD spread. This suggests that relying solely on the number of infected pine trees to evaluate prevalence severity within a sub-compartment is insufficient and does not fully capture the complexity of the PWD prevalence status. Consequently, a standard diagrammatic scale based on the *E* value has been employed to evaluate the PWD prevalence at the sub-compartment scale.

This study, of the first time, combined the number of infected trees and the area of forest sub-compartments to compare baseline accuracy for pine wilt disease (PWD) prevalence. The analysis revealed significant variations in the distributions of data across different prevalence parameters: *N*, *NC*, Ln (*N*), and Ln (*NC*). The pronounced leftward skew and high kurtosis observed in the distributions of *N* and *NC* highlight the complexity and variability of PWD prevalence at the sub-compartment scale (Belan et al., 2014; Perina et al., 2019). The logarithmic transformations Ln (*N*) and Ln (*NC*) normalized the skewness, suggesting these transformations can mitigate the impact of extreme values and provide a more balanced representation of the prevalence data. Among these, Ln (*NC*) was selected as the baseline accuracy metric due to its optimal normal distribution characteristics, which are crucial for reliable statistical analysis and modeling of PWD prevalence. This approach offers a more nuanced understanding of PWD distribution, potentially leading to more effective management strategies (Chiang et al., 2017).

Semi-quantifying incomprehensible or intuitive data on an ordinal scale can provide a more accurate representation of the disease occurrence (Barbedo, 2016; Chiang and Bock, 2022). The results of Chiang et al. (2014) indicated that an amended 10% category scale with additional grades at low severity (also known as ‘Nearest percent estimates’) can be considered a superior choice for evaluating disease severity when the use of a scale is preferred over the Horsfall–Barratt scale (Bock et al., 2010). Our findings demonstrate that by categorizing Ln (*NC*) values into 12 levels and establishing *DS* scales, the *DS* provides a standardized framework for evaluating the severity of PWD prevalences within sub-compartments. Moreover, the 12-level scale was chosen to strike a balance between providing sufficient detail and maintaining practical usability for forest managers. It offers more granularity than many existing scales while remaining manageable for field application.

Different regions may experience variations in pine sawyer beetle population density, life cycle timing, dispersal patterns, and host preferences (Zhou et al., 2024; Futai and Ishiguro, 2025). These factors could significantly influence the spatial and temporal patterns of PWD spread, potentially impacting the accuracy and interpretation of the *DPI* method. For instance, areas with higher beetle populations or more favorable conditions for beetle activity might experience more rapid or extensive PWD spread, which could be reflected in higher *DPI* values or more rapid changes in *DPI* over time. Moreover, the application of *DPI* at different geographic scales, including town and county levels, demonstrates the versatility and applicability of the assessment framework. The dynamic prevalence of PWD at the county level and changes in *DPI* across different years highlight the spatial and temporal variability of the prevalence. The case studies of Lin’an District and THY Town illustrated the effectiveness of *DPI* in capturing the complexity of the PWD prevalence at various geographic scales. The observed decreases in *DPI* values over time suggest a potential trend of epidemic mitigation, which warrants further investigation.

Furthermore, the *DPI* index was developed to evaluate PWD prevalence in this study, analogous to the plant disease index (Barbedo, 2016). Analysis of the PWD prevalence from 2021 to 2023 utilizing the novel index *DPI* revealed a consistent pattern of normal distribution characteristics in the *DS* values across different years. The introduction of *DPI* enhances the comprehensiveness of the assessment framework by summarizing the occurrence of PWD across the region. The observed changes in *DPI* over the three-year period indicate a potential trend of epidemic mitigation. These observed changes suggest a potential trend of epidemic mitigation, indicating that the current control measures may be having a positive impact on PWD prevalence in the region. Our analysis demonstrated the effectiveness of DPI in capturing the complexity of PWD prevalence at various geographic scales, including the county level. This county-level variability in DPI values over time suggests spatial and temporal differences in PWD prevalence, which could be influenced by factors such as local environmental conditions and management practices. The dynamic trends in *N*, *P*, and *DPI* are not merely statistical artifacts but evidence of a functioning PWD integrated management framework (Chen and Li, 2024).

## 5 Conclusion

In conclusion, this study introduced a novel algorithm that provides a standardized framework for evaluating PWD prevalence at the sub-compartment scale and across various geographic levels. The development of *DS* and *DPI* underscores the temporal and spatial heterogeneity of PWD prevalence patterns across sub-compartments. Future work will focus on enhancing the application of *DPI* to improve PWD prevalence monitoring and management.

## Data Availability

The original contributions presented in the study are included in the article/Supplementary Material. Further inquiries can be directed to the corresponding author.
